# MMP-3 plays a major role in calcium pantothenate-promoted wound healing after fractional ablative laser treatment

**DOI:** 10.1007/s10103-021-03328-8

**Published:** 2021-05-14

**Authors:** Sebastian Huth, Laura Huth, Yvonne Marquardt, Maria Cheremkhina, Ruth Heise, Jens Malte Baron

**Affiliations:** 1 Department of Dermatology and Allergology, Medical Faculty RWTH Aachen University, Pauwelsstrasse 30, 52074 Aachen, Germany; 2 Interdisciplinary Center for Laser Medicine, Medical Faculty RWTH Aachen University, Aachen, Germany

**Keywords:** 3D skin model, Wound healing, MMP-3, Calcium pantothenate, Dexpanthenol

## Abstract

Ablative fractional laser treatment leads to a loss of matrix metalloproteinase-3 (MMP-3) expression; therefore, in the present in vitro study, we addressed the role of MMP-3 and its regulation by calcium pantothenate in wound healing processes at the molecular level. Utilizing confocal laser microscopy, we investigated MMP-3 protein expression in fractional ablative CO_2_ laser-irradiated skin models. In addition, we established full-thickness 3D skin models using fibroblasts and keratinocytes with a MMP-3 knockdown that were irradiated with a fractional ablative Er:YAG laser to set superficial injuries with standardized dimensions and minimal thermal damage to the surrounding tissue. We revealed an upregulation of MMP-3 protein expression in laser-irradiated skin models receiving aftercare treatment with calcium pantothenate. Skin models with MMP-3 knockdown exhibited a slower wound closure after laser treatment compared to controls. Gene expression profiling detected an MMP-3 knockdown-dependent upregulation of cytokines and chemokines (e.g. IL-36B, CXCL17, IL-37, CXCL5), antimicrobial peptides (e.g., S100A7, S100A12), epidermal crosslinking enzymes (TGM5), and differentiation markers (e.g., LOR, KRT1, FLG2). We also detected a downregulation of cathepsin V and MMP-10, both of which play a prominent role in wound healing processes. After fractional ablative laser injury, an aftercare treatment with calcium pantothenate accelerated wound closure in MMP-3 expressing models faster than in MMP-3 knockdown models. Our data substantiate a major role of MMP-3 in wound healing processes after ablative laser treatments. For the first time, we could show that calcium pantothenate exerts its wound healing-promoting effects at least partly via MMP-3.

**Supplementary Information:**

The online version contains supplementary material available at 10.1007/s10103-021-03328-8.

## Introduction

Following injury, the process of wound healing is broadly classified into three different phases, namely inflammation, proliferation, and remodeling [1]. During these phases, nearly all cell types express distinct patterns of matrix metalloproteinases (MMPs) [2]. MMPs are a family of endopeptidases which are involved in the degradation of extracellular matrix (ECM) components, thereby regulating various physiological processes [3]. Members of the MMP family exhibit a prominent role in wound healing by modifying the wound matrix, thus enabling cell migration and tissue remodeling, which is crucial for wound re-epithelialization [4]. During wound healing, expression and activity of different MMPs are precisely regulated [4]. Among all MMP family members, MMP-3 (also known as stromelysin-1) features a distinctive expression pattern during wound healing [5]. The functional role of MMP-3 is the degradation of collagens II, III, IV, IX, and X, as well as proteoglycans, laminin, and fibronectin [4]. Former studies have pointed to a pivotal role of MMP-3 in wound healing; e.g., patients with impaired wound healing showed a decreased expression of MMP-3 [6] and MMP-3 knockout mice suffered from delayed excisional wound healing due to a failure in wound contraction [7].

So far, data on molecular processes affected by ablative laser treatments are still scarce. Previously, we performed a transcriptomic microarray profiling on day 5 after laser treatment in 3D skin models irradiated with 100 mJ/cm^2^ using a fractional ultrapulsed CO_2_ laser and identified a downregulation of various MMPs including MMP-3 [8]. While qRT-PCR experiments confirmed the decrease of MMP-3 mRNA expression in 3D skin models on day 5 after the ablative CO_2_ laser treatment, independent experiments detected an upregulation of MMP-3 expression in laser-irradiated skin models following aftercare treatment with calcium pantothenate [8, 9].

An optimized wound care is an important goal in order to achieve a rapid wound closure without complications. In this context, preparations containing ingredients that help to promote wound healing are of particular interest. In our previous study, we detected that the addition of calcium pantothenate or dexpanthenol to laser-injured 3D skin models resulted in a significantly faster wound closure compared to control models, which was associated with the increased expression of MMP-3 [9]. Calcium pantothenate (vitamin B5) is the precursor for the biosynthesis of coenzyme A (CoA), which is a fundamental enzyme cofactor in a vast number of metabolic and biosynthetic processes [10–12].

Although MMP-3 appears to play an important role in wound healing, our previous data showed a downregulation of MMP-3 after laser irradiation of 3D skin models that could be rescued by calcium pantothenate treatment [8, 9]. Therefore, the aim of the present in vitro study was to investigate the role of MMP-3 in the wound healing process after ablative fractional laser treatment and its regulation by calcium pantothenate using laser-injured 3D skin models.

## Materials and methods

### Isolation and cell culture of normal human epidermal keratinocytes (NHEK) and normal human dermal fibroblasts (NHDF)

NHDF and NHEK cells were isolated and cultivated as described previously [8, 9, 13].

### MMP-3 knockdown in NHDF and NHEK

NHDF and NHEK cells with a transient MMP-3 knockdown were generated using the MMP-3 human siRNA Oligo Duplex (SR302926; Origene Technolgies, Rockville, Maryland, USA), according to the manufacturer’s instructions. Transient lipofection on NHDF and NHEK cells was done with Lipofectamine 2000 (Thermo Fisher, Langenselbold, Germany). As controls, cells were transfected with scramble negative control siRNA duplex (Origene).

### 3D skin equivalents and laser irradiation

Full-thickness 3D skin equivalents were established as described previously [9]. In brief, the dermal part of the skin equivalents was constructed by merging bovine collagen I solution (Vitrogen, Cohesion Technologies, Palo Alto, CA, USA) and × 10 concentrated Hank’s balanced salt solution (Gibco/Invitrogen, Darmstadt, Germany) (ratio 8:1). After neutralization with 1M NaOH, 1 × 10^5^ NHDF cells were added and poured into polycarbonate cell culture inserts (3 μm pore size, Nunc; Thermo Fisher)_._ After 2 days of incubation at 37 °C and 5% CO_2_, 2 × 10^6^ NHEK cells were seeded on the dermal equivalent. On the following day, skin equivalents were lifted to the air-liquid interface (ALI). Laser treatment of skin models was performed by fractional ablative CO_2_ (ultrapulse, CPG handpiece, 1.3-mm spot size, 100 mJ, 100 Hz, scan pattern density 55%, Lumenis, Israel) or Er:YAG (MCL31 Dermablate, fluence per pulse 10 J/cm^2^, 6 pulses (60 J total fluence), 4 Hz, pulse duration 300 μs, coverrate 10%, 60 J, Asclepion, Germany) laser. Experiments were performed three times independently in duplicates.

### Light microscopy

For light microscopy, 4-μm cryosections of 3D skin models embedded in Tissue-Tek O.C.T. ™ compound (Sakura Finetek) were stained with hematoxylin and eosin (H&E) and subsequently examined by a photomicroscope (DMIL, Leitz, Wetzlar, Germany).

### Immunofluorescence analysis by confocal laser scanning microscopy

For immunofluorescence, 10-μm cryosections of skin models embedded in Tissue-Tek O.C.T.™ were fixed for 10 min in acetone at 4 °C. First antibody MMP-3 (HPA007875, Atlas Antibodies, Stockholm, Sweden) was diluted with antibody diluent (Dako, Glostrup, Denmark) and incubated at room temperature for 1 h. Following three washing steps with PBS, the sections were incubated in fluorochrome-conjugated secondary antibody Alexa Fluor 488 IgG H+L (Molecular Probes, Eugene, OR, USA) for 1 h at room temperature. Subsequently, the sections were washed three times with PBS, and cell nuclei were stained with DAPI (Applichem, Darmstadt, Germany). After a final washing step, sections were mounted in fluorescent mounting medium (Dako) and coverslipped. The sections were stored in the dark at 4 °C. Microscopy was performed with a laser scanning confocal microscope (LSM 710; Carl Zeiss, Oberkochen, Germany).

### RNA-isolation and quantitative real-time PCR analysis

Total RNA from 3D skin models was extracted with the Nucleo Spin RNA Kit (Macherey and Nagel, Düren, Germany), according to the manufacturer’s instructions. RNA was quantified using photometric measurement (NanoDrop Technologies, Wilmington, DE, USA), and its integrity was analyzed on a 2100 bioanalyzer (Agilent Technologies, Palo Alto, CA, USA). Purified RNA was reverse-transcribed into cDNA using SS VILO Mastermix (Life Technologies, Langenselbold, Germany). For qRT-PCR analyses, an ABI PRISM 7000 Sequence Detection System (Applied Biosystems, Weiterstadt, Germany) was used with assay-on-demand gene expression products for IL36B (Hs00758166_m1), LOR (Hs01894962_m1), KRT1 (Hs01549614_g1), FLG2 (Hs00418578_m1), S100A12 (Hs00194525_m1), MMP-3 (Hs00233962_m1), and HPRT (Hs99999909_m1). All measurements were performed in triplicate in separate reaction wells.

### Analysis of gene expression using microarray analysis

To generate amplified sense-strand cDNA, 300 μg purified mRNA of each model were processed using the WT Expression Kit (Ambion, Austin, TX, USA). Fragmentation and labeling were done with the Affymetrix® GeneChip® WT Terminal Labeling Kit Assay (Affymetrix, Inc., Santa Clara, CA, USA). Hybridization cocktails were prepared and applied to Clariom™ S assay (Thermo Fisher Scientific). Hybridization was performed in 45 °C oven at 60 rpm for 16 h. Using the GeneChip® Fluidics Station 450 the hybridized Clariom™ S assays were washed and stained (Fluidics Protocol FS450_0001). Arrays were scanned using Affymetrix GeneChip® Scanner 3000 7G controlled by GeneChip® Operating Software (GCOS) version 1.4 to produce CEL intensity files. Expression values of each probe set were determined, and MMP-3 knockdown models were compared to MMP-3 expressing controls using the GeneSpring GX 14.9 software (Agilent Technologies, Frankfurt am Main, Germany). Normalization method: Quantile.

#### **Statistical analysis**

Data are given as arithmetical means ± standard deviation (SD). Mann-Whitney *U* test was performed with GraphPad PRISM version 7 (La Jolla, CA, USA). Values of *p* < 0.05 were considered statistically significant.

## Results

**Fig. 1:**
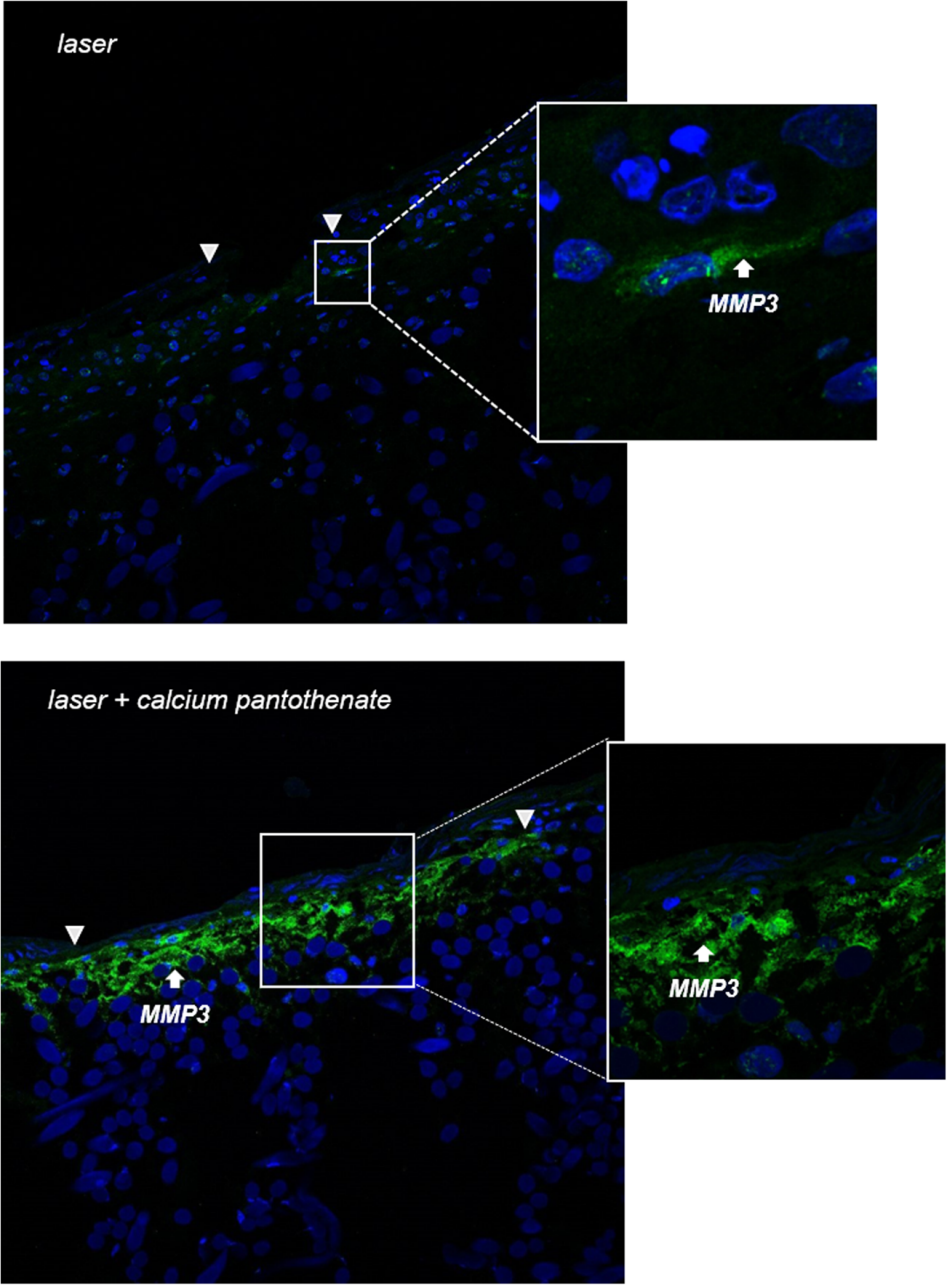
Aftercare treatment of ablative laser injury with calcium pantothenate upregulates the protein expression of MMP-3. Microscopy was performed with a laser scanning confocal microscope on day 3 after non-sequential fractional ultrapulsed CO_2_ laser treatment (CPG handpiece, 1.3 mm spot size, 100 mJ, 100 Hz, scan pattern density 55%). Representative images of three independent experiments performed in duplicates are shown. White arrows indicate the margins of the laser lesion

In a first set of experiments, we examined the protein expression of MMP-3 in 3D skin models that were injured with a fractional ablative CO_2_ laser and compared the expression pattern with an approach that was additionally treated with calcium pantothenate as aftercare (Fig. 1). Using confocal laser scanning microscopy, we detected an increased expression of MMP-3 close to the basement membrane in 3D skin models that were additionally treated with calcium pantothenate compared to only laser-treated models.

**Fig. 2:**
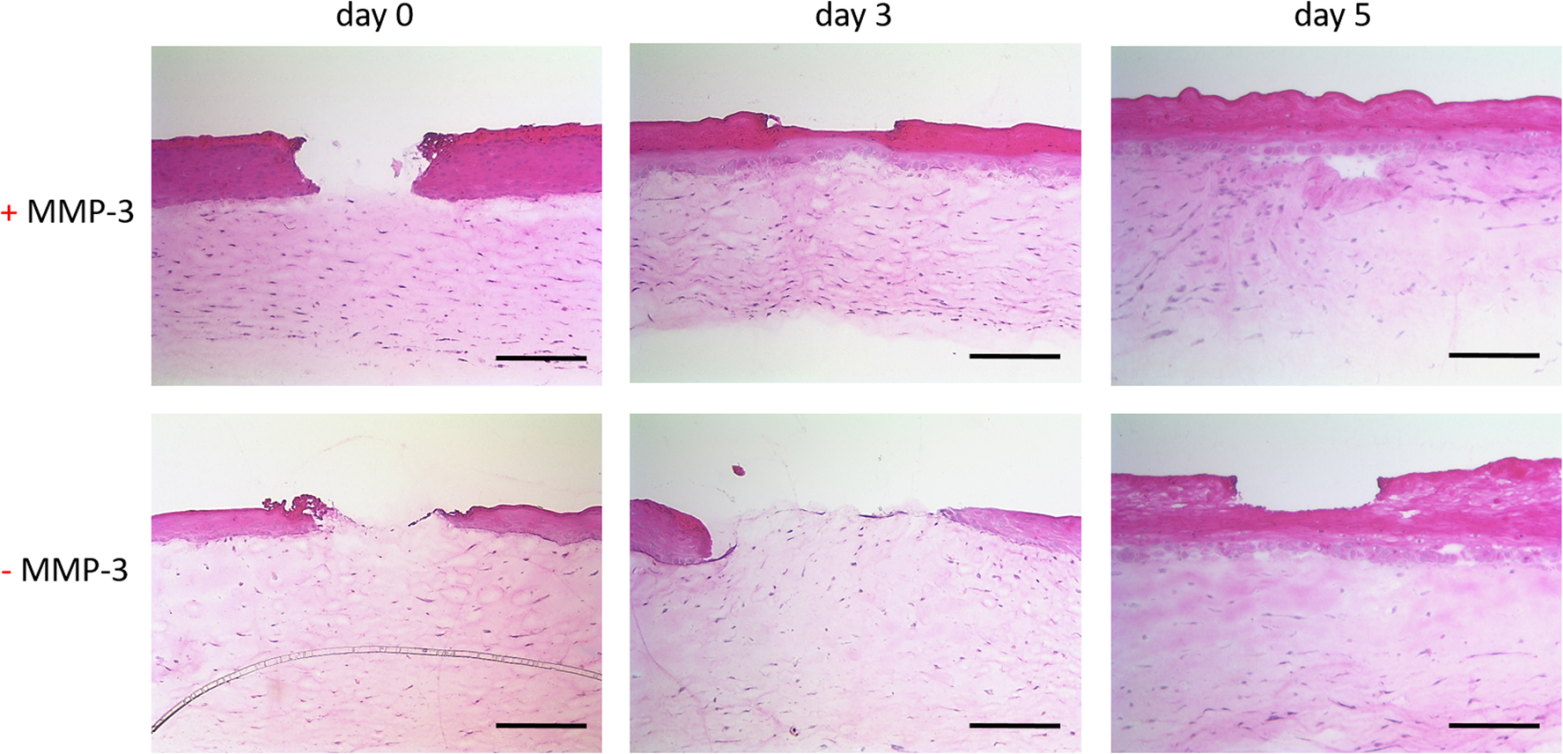
MMP-3 knockdown delays wound healing. Representative HE stained sections of fractional Er:YAG laser irradiated (total fluence 60 J, 6 pulses, 4 Hz, pulse duration 300 μs, coverrate 10%) 3D skin models either comprising MMP-3 expressing cells (+MMP-3) or MMP-3 knockdown cells (-MMP-3) at different timepoints after treatment. Representative images of three independent experiments performed in duplicates are shown. Magnification = × 100, scale bar = 200 μm

To address the role of MMP-3 in wound healing in more detail, we established epidermal keratinocytes and dermal fibroblasts with a siRNA-mediated knockdown of MMP-3 (Fig. S1). To investigate the impact of MMP-3 on wound closure, we used an Er:YAG laser to set superficial injuries with standardized dimensions and minimal thermal damage to the surrounding tissue in our model systems. Setting injuries with an Er:YAG laser in 3D skin models that were generated with the MMP-3 knockdown cells, the models exhibited a slower wound closure on day 3 and 5 after laser treatment compared to models generated with MMP-3 expressing cells (scrambled control) (Fig. 2). While MMP-3 expressing models showed advanced healing on day 3 after laser injury, the MMP-3 knockdown models still exhibited a pronounced laser wound with a completely ablated epidermal part. Skin models that comprised MMP-3 expressing cells showed a completely restored epithelial part on day 5 after laser treatment, in contrast to MMP-3-deficient models.

**Fig. 3:**
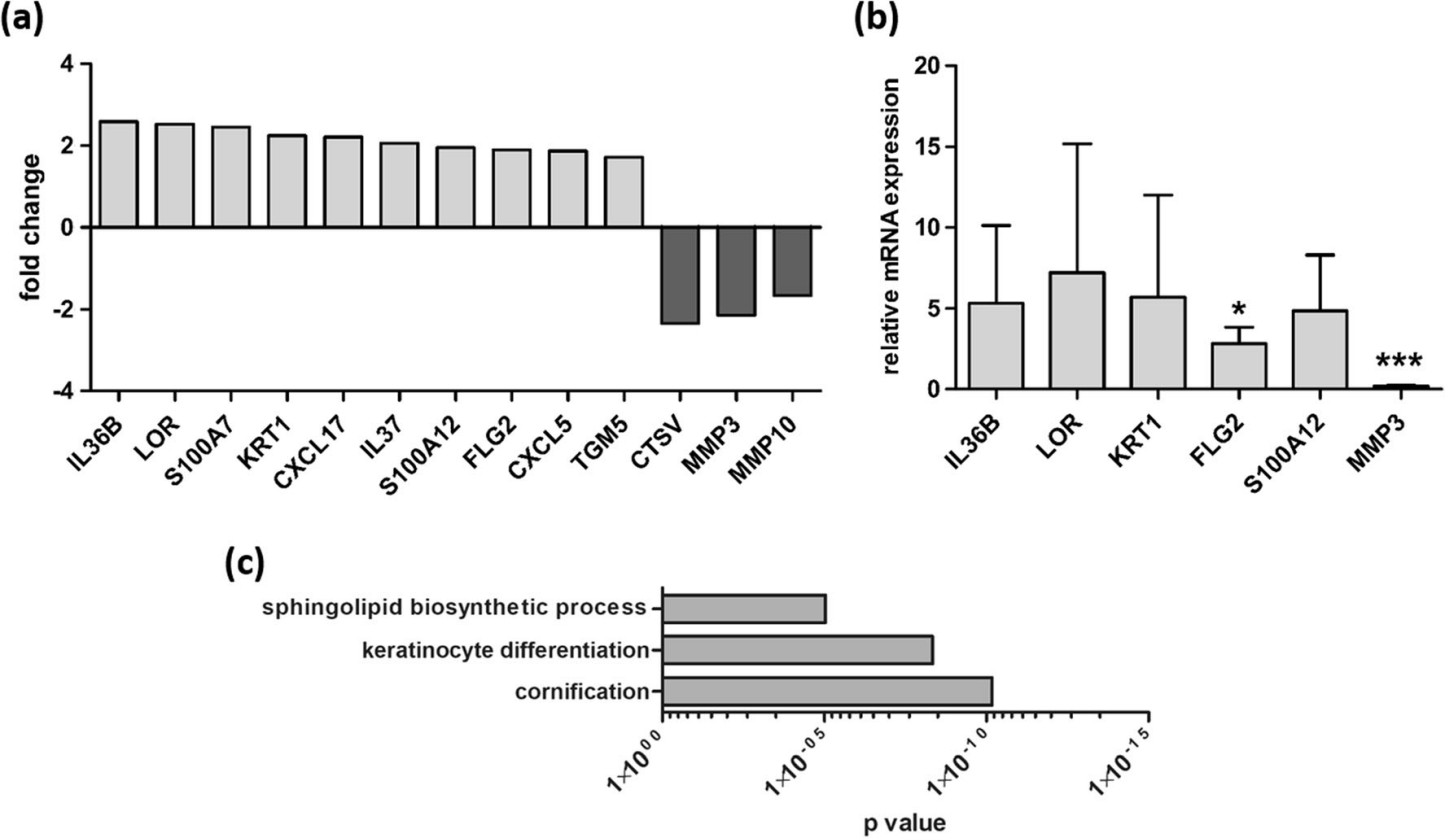
MMP-3 knockdown provokes a gene expression pattern of delayed wound healing mechanisms. (**a**) Representative microarray analysis revealed differentially expressed genes in MMP-3 knockdown models in comparison to MMP-3 expressing controls. (**b**) TaqMan qRT-PCR analysis of selected genes. Mean values of three independent experiments performed in triplicate are shown. Vertical lines: + standard deviation (SD). *p* values are significant if < 0.05. *p < 0.05, ***p < 0.001. (**c**) Gene ontology (GO) analysis of microarray results of MMP-3 knockdown models in comparison to MMP-3 expressing controls

In a next step, transcriptomic microarray profiling was carried out to assess the molecular effects of MMP-3 on wound healing (Fig. 3a). We detected an upregulation of differentiation markers (e.g., LOR, KRT1, FLG2), cytokines and chemokines (e.g.. IL-36B, CXCL17, IL-37, CXCL5), epidermal crosslinking enzymes (TGM5), and antimicrobial peptides (e.g., S100A7, S100A12) in laser-irradiated skin models with a MMP-3 knockdown. On the other side, we found a downregulation of collagen hydrolyzing cathepsin V and MMP-10. In addition, qRT-PCR analyses confirmed the microarray results for selected genes (Fig. 3b). Using a gene set comparison analysis, gene ontology (GO) annotations revealed an association of MMP-3 knockdown in laser-injured models with categories like “cornification”, “keratinocyte differentiation”, and “sphingolipid biosynthetic process” (Fig. 3c).

**Fig. 4:**
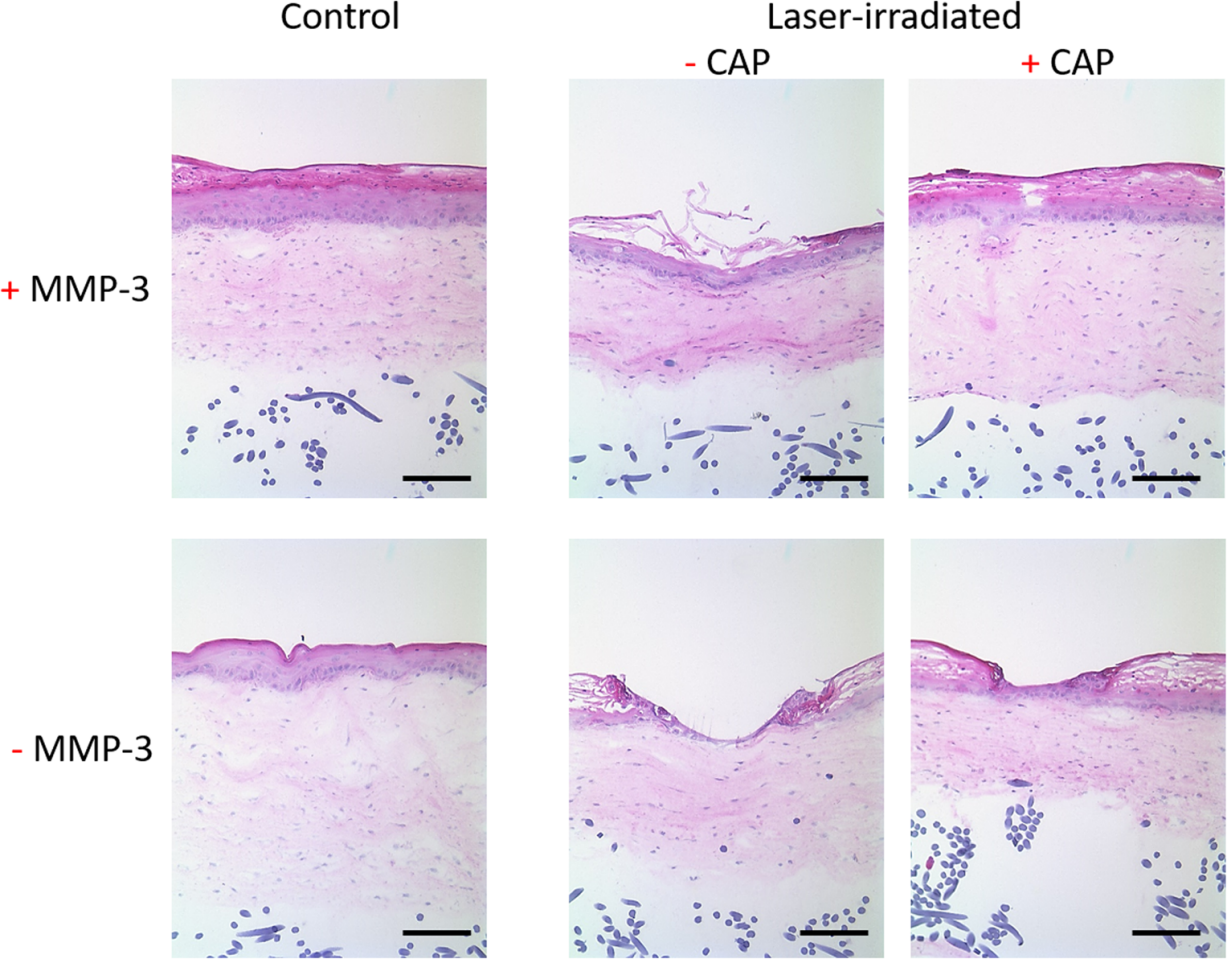
Calcium pantothenate promotes wound healing effects via MMP-3. Representative HE stained sections of untreated controls and Er:YAG laser irradiated (total fluence 60 J, 6 pulses, 4 Hz, pulse duration 300 μs, coverrate 10%) 3D skin models either comprising MMP-3 expressing cells (+MMP-3) or MMP-3 knockdown cells (-MMP-3) on day 3 after laser treatment. One approach was treated with calcium pantothenate (+CAP), while the other one was not (-CAP). Representative images of three independent experiments performed in duplicates are shown. Magnification = × 100, scale bar = 200 μm. CAP = calcium pantothenate

After we had shown a stimulating effect of calcium pantothenate on MMP-3 expression, we wanted to investigate whether calcium pantothenate-mediated wound healing effects are MMP-3 dependent (Fig. 4). MMP-3 expressing models that were treated with calcium pantothenate showed a fully restored epidermal part on day 3 after laser irradiation. In contrast, laser-injured MMP-3 knockdown models still exhibited an epidermal lesion when treated with calcium pantothenate.

## Discussion

Cutaneous wound healing is a complex multi-step process including different overlapping phases: inflammation, proliferation, and remodeling [1]. Different cellular and molecular processes with a variety of modulators, including MMPs, are essential for successful wound closure [14]. MMPs are a family of zinc-dependent endopeptidases that can be activated and regulated by a variety of hormones, growth factors, cytokines, and physicochemical agents as well as by the counteracting actions of endogenous tissue inhibitors of metalloproteinases (TIMPs) [15]. Nearly two decades ago, it was shown that MMP-3 is induced by dermal fibroblasts and basal keratinocytes during wound repair [16]. Since then, data from several studies have pointed to a central role of MMP-3 in wound healing. A study by Utz and colleagues detected that MMP-3 expression is reduced in patients with impaired wound healing [6]. Furthermore, Bullard et al. exhibited that mice with a targeted deletion in the MMP-3 gene suffer from delayed excisional wound healing due to a failure in wound contraction [7]. Additionally, they could demonstrate that MMP-3 is responsible for the contraction of fibroblasts and initiates wound contraction [17]. Today, it is known that MMP-3 together with MMP-1 and MMP-9 is one of the most important chemokine regulators in wound healing [4]. Interestingly, using a laser-injured standardized human 3D skin model, we previously detected a downregulation of MMP-3 expression on day 5 after fractional ablative laser treatment [8] and, on the other hand, an upregulation of MMP-3 expression after additional treatment with dexpanthenol or its salt calcium pantothenate [9]. These observations led us to further examine the role of MMP-3 in wound healing processes after ablative fractional laser treatment and the regulatory effects of calcium pantothenate on MMP-3 expression in more detail.

Our previous studies have proven that laser irradiation of human 3D skin models facilitates the setting of multiple injuries with defined dimensions, thus enabling a standardized investigation of wound healing on the molecular and histological level [8, 9, 18–20]. In our present study, we utilized laser-irradiated 3D wound healing skin models to investigate the role of MMP-3 in post-laser wound healing processes and the regulatory effects of calcium pantothenate on MMP-3 expression on the molecular level. The skin expresses negligible amounts of MMP-3 under resting conditions, while the expression of MMP-3 in dermal fibroblasts and basal keratinocytes is upregulated during wound repair [16, 21, 22]. Using confocal laser scanning microscopy, we were able to detect upregulated MMP-3 protein expression in laser-irradiated skin models that were treated with calcium pantothenate – in both the epidermal and dermal part of the model, adjacent to the basement membrane. However, performing in vitro studies, we previously demonstrated that the expression of MMP-3 decreases in 3D skin models on day 5 after ablative laser irradiation [8]. Our new data indicate that this decreased expression of MMP-3 on day 5 after laser injury can be rescued by an aftercare treatment with calcium pantothenate. Such data can help to improve wound care and our currently sparse molecular knowledge of post-laser treatment.

To further investigate the role of MMP-3 in wound healing, we established fibroblasts and keratinocytes with a stable MMP-3 knockdown. Previous studies showed that a reduced or missing MMP-3 expression is associated with an impaired and delayed wound healing [6, 7, 17]. In agreement, we could clearly attribute a delayed wound closure with lacking MMP-3 expression in our laser-irradiated 3D wound healing skin models, which confirms that MMP-3 plays an important role in wound healing. Our next step was to study the molecular mechanisms of action of MMP-3 in more detail.

Gene expression profiling revealed an upregulation of various differentiation markers and transglutaminase 5 (TGM5), which is described to catalyze crosslinking of structural proteins, thereby inducing epidermal cornification [23]. These data possibly reflect a lower amount of proliferating keratinocytes in laser-injured MMP-3 knockdown models and indicate a role for MMP-3 in cell proliferation during wound repair. These findings were substantiated by a GO analysis showing an association of MMP-3 knockdown in laser-injured models with categories like “cornification” and “keratinocyte differentiation”.

Finding an upregulation of pro-inflammatory cytokines, chemokines, and antimicrobial peptides in MMP-3 knockdown models supports the previously postulated anti-inflammatory effects of MMP-3 [24]. On the other side, we detected a downregulation of cathepsin V (CTSV) and MMP-10. While previous studies have shown that cathepsins are involved in a variety of physiological processes, including a role in wound healing [25], MMP-10 is strongly expressed in keratinocytes at the wound edge and contributes to proliferation and differentiation processes during wound healing [26]. A MMP-3 knockdown-dependent downregulation of these two genes further emphasizes the wound healing-promoting effects of MMP-3 at the molecular level. Our molecular data help to better understand the role of MMP-3 in wound healing specifically after treatment with ablative laser systems.

Dexpanthenol, the stable alcoholic analog of calcium pantothenate, is widely used in dermatological therapies. However, its diverse mechanisms of action are still not fully understood. Detecting a stimulatory effect of calcium pantothenate on MMP-3 expression, our next step was to investigate whether the effects of calcium pantothenate on wound healing are MMP-3 dependent. Interestingly, the addition of calcium pantothenate accelerated the wound closure in MMP-3 expressing models more significant than in MMP-3 knockdown models, clearly indicating that calcium pantothenate exerts its clinical wound healing effects via MMP-3. The fact that MMP-3 knockdown models treated with calcium pantothenate showed a more advanced wound healing than MMP-3 knockdown models that were not treated with calcium pantothenate could be explained by the fact that we only induced a siRNA-mediated knockdown and not a complete knockout of the gene. Furthermore, it is known that calcium pantothenate supports wound healing in human skin tissue by other mechanisms such as the upregulation of heme oxygenase-1 (HMOX-1) in dermal fibroblasts. HMOX-1 is known to reduce oxidative stress, attenuate inflammatory responses, and lower the rate of apoptosis [27]. In addition, it has been shown that calcium pantothenate modulates gene expression and proliferation in human dermal fibroblasts [28].

In conclusion, we present an in vitro study investigating the effects of MMP-3 in wound healing processes after fractional ablative laser treatment and its regulation by calcium pantothenate using laser-injured 3D skin models. To our knowledge, we present the first study showing that MMP-3 is an essential factor for the wound healing effects of calcium pantothenate. Our data, on the one hand, substantiate the key role of MMP-3 in wound healing processes of the skin after fractional ablative laser treatment and, on the other hand, indicate that calcium pantothenate exerts its clinical effects at least partly via MMP-3. Our study helps to better understand the role of MMP-3 in wound healing specifically after treatment with ablative laser systems and how calcium pantothenate exerts its wound healing effects. Moreover, our results contribute to the currently sparse molecular knowledge of post-laser wound healing processes and treatment and can help to improve wound care.

## Supplementary Information

Fig. S1 Quantitative real-time PCR analysis of skin models comprising MMP-3 expressing cells (+MMP-3) and MMP-3 knockdown cells (-MMP-3). The relative MMP-3 mRNA levels were normalized to HPRT rRNA. Relative MMP-3 expression levels from two representative models (1 +MMP-3 and 1 -MMP-3) are shown.

High resolution image (TIF 533 kb)
