# Supplementary figures and images for: MMP-3 plays a major role in calcium pantothenate-promoted wound healing after fractional ablative laser treatment
Source: Lasers Med Sci. 2021 May 14;37(2):887–94. doi: 10.1007/s10103-021-03328-8 (PMC8918166; doi:10.1007/s10103-021-03328-8)

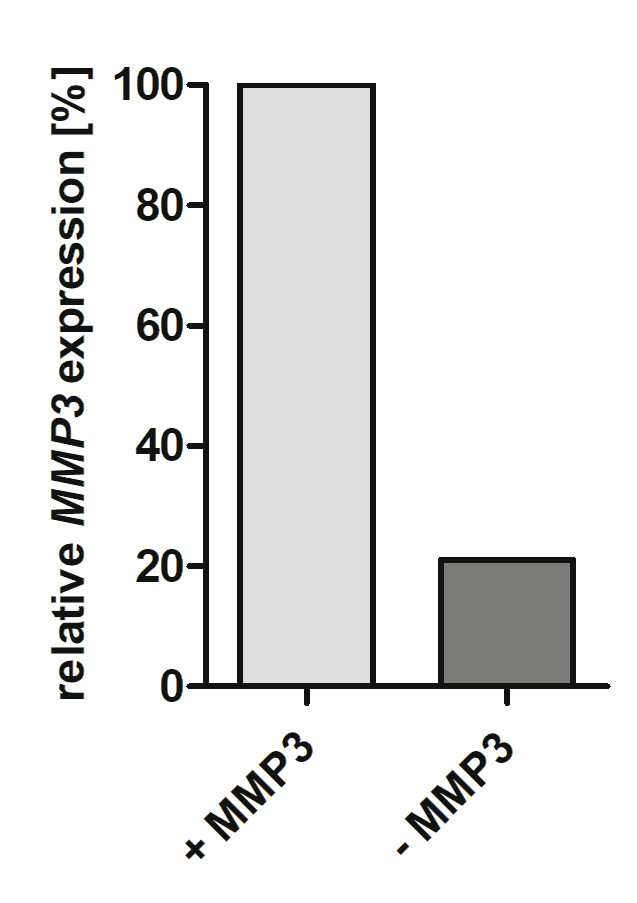

Supplement: Supplementary file 1 — Quantitative real-time PCR analysis of skin models comprising MMP-3 expressing cells (+MMP-3) and MMP-3 knockdown cells (-MMP-3). The relative MMP-3 mRNA levels were normalized to HPRT rRNA. Relative MMP-3 expression levels from two representative models (1 +MMP-3 and 1 -MMP-3) are shown. [file 10103_2021_3328_Fig5_ESM.png]

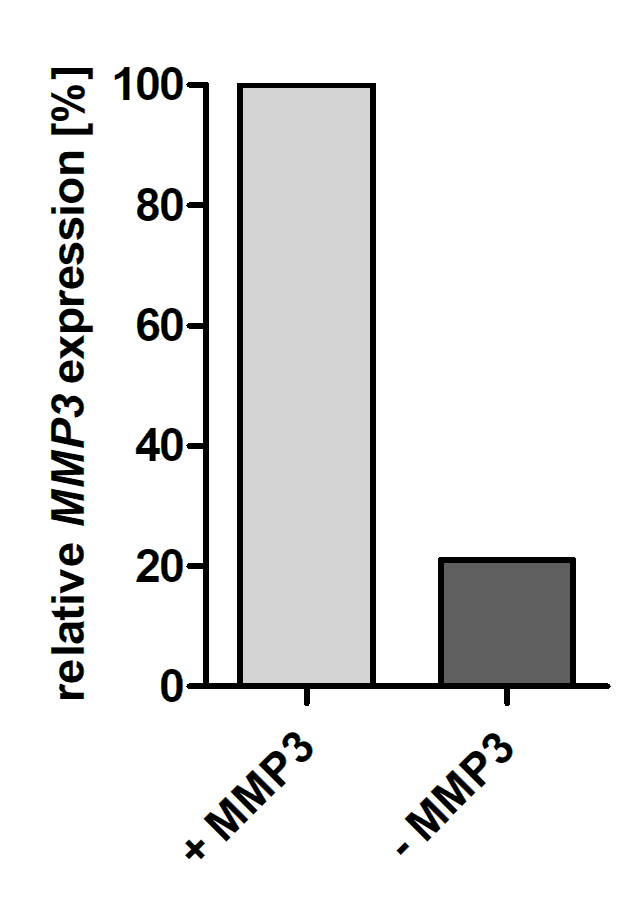

Supplement: Supplementary file 2 — High resolution image (TIF 533 kb) [file 10103_2021_3328_MOESM1_ESM.tif]
